# Electrophysiological Characterization of Murine Vestibular Efferent Neurons and Modulation by Acute Peripheral Vestibular Deprivation

**DOI:** 10.1007/s12264-025-01502-4

**Published:** 2025-09-18

**Authors:** Jinyu Wang, Mengfan Xu, Lei Zhang, Wenjie Liu, Siyue Wang, Liqin Wang, Ning Cong, Geng-Lin Li, Jing Wang

**Affiliations:** 1 ENT Institute and Department of Otorhinolaryngology, Eye and ENT Hospital, Fudan University, Shanghai, 200031 China; 2 NHC Key Laboratory of Hearing Medicine Research, Shanghai, 200031 China; 3 Institutes of Brain Science, State Key Laboratory of Medical Neurobiology, and MOE Frontiers Center for Brain Science, Fudan University, Shanghai, 200031 China

**Keywords:** Vestibular efferent neuron, Acute unilateral peripheral vestibular dysfunction, A-type potassium current, Vestibular compensation

## Abstract

Vestibular efferent neurons in the brainstem provide direct cholinergic innervation to peripheral vestibular organs, thereby modulating their sensory responsiveness. In this study, a genetically targeted mouse model with choline acetyltransferase-driven fluorescent labeling enabled the precise localization of these neurons to the dorsolateral region of the genu of the facial nerve. Whole-cell patch-clamp recordings in acute brainstem slices revealed that virtually all neurons exhibited spontaneous action potential firing, with marked heterogeneity in discharge patterns and after-hyperpolarization kinetics. Prominent A-type potassium currents were identified and found to be differentially regulated by acetylcholine and calcitonin gene-related peptide. Acute unilateral vestibular deprivation induced a bilateral enhancement of spontaneous firing, indicating sensitivity to altered sensory input. These findings define the intrinsic electrophysiological properties and neuromodulatory mechanisms of vestibular efferent neurons, providing mechanistic insight into their roles in both physiological regulation and adaptive plasticity within the vestibular system.

## Introduction

The vestibular system detects head motion and orientation to coordinate vital reflexes, which are crucial for maintaining postural balance and controlling gaze to stabilize vision [1, 2]. Furthermore, more and more studies published recently have started to reveal engagement of the vestibular system in high-level sensorimotor functions, including perceptual judgment, motor decision-making, and motor and learning ability [3, 4]. The vestibular system comprises peripheral end organs and nuclei located in the brainstem, cerebellum, thalamus, and cerebral cortex, where many of these nuclei integrate sensory information from the periphery and coordinate vital reflexes [5, 6]. The vestibular system begins with hair cells in the inner ear, which generate electrical signals in response to head movements. These signals are transmitted *via* the vestibular afferent nerve to the vestibular nuclei in the brainstem and subsequently relayed to the cerebellum, thalamus, and cerebral cortex for the integration and regulation of balance, posture, and spatial orientation [7]. The efferent vestibular system (EVS) originates from the efferent vestibular nucleus (EVN) located in the brainstem. The focus of this study was the EVN, which is comprised of vestibular efferent neurons (VENs) that establish synaptic connections with peripheral vestibular organs and other related systems [2, 8, 9].

The functional role of the EVN has been a long-standing mystery primarily due to the limited number of neurons and their relatively sparse distribution. Cullen’s review [10] concluded that the mammalian EVN plays a crucial role in calibrating and safeguarding the functional efficacy of vestibular circuits over longer-time courses. Mathews proposed that the EVN is associated with efference copy generated from spinal cord circuitry during locomotion and participates in vestibular plasticity and compensation mechanisms [11].

To date, studies involving direct recordings from mouse VENs have been limited. David et al. used transgenic mice expressing fluorescent proteins under the choline acetyltransferase (ChAT) promoter to identify cholinergic VENs for the more accurate location of VENs [12]. Applying patch-clamp techniques, they conducted preliminary investigations into the basic electrophysiological properties of these neurons, focusing on passive membrane characteristics, firing patterns, and synaptic input profiles [13, 14].

However, their studies were constrained by the small number of recorded neurons, which precluded detailed analyses of their spontaneous activity and more comprehensive cellular classification. In addition, their work only addressed the electrophysiological characteristics of VENs under normal physiological conditions, leaving unexplored how these neurons behave in pathological contexts. Moreover, while Leijon’s study identified the expression of Kv4.2 and Kv4.3, mediating A-type K^+^ currents, further investigation into the regulation of these currents in VENs remains lacking.

Voltage-dependent K^+^ (Kv) channels are extensively documented to be expressed in the brain. These channels are involved in modulating neuronal excitability, contribute to rapid spiking activity, and exert control over neural networks [15–17]. Among the Kv4 α-subunits, Kv4.2 and Kv4.3 are the primary subunits responsible for generating the somatodendritic A-type K^+^ currents (I_A_) in the central nervous system [18, 19]. I_A_ is a rapidly-activating, subthreshold outward current that has been identified to be an important determinant in shaping the fast repolarization of neuron action potentials and repetitive firing properties [20, 21]. The modulation of I_A_ is considered to be a critical regulator of neuronal excitability.

As pivotal components of the EVS, VENs exert a significant influence on the overall functionality of the vestibular neural pathway. While there is a consensus on the location of the EVN, its distinct role and regulatory mechanisms have yet to be established due to the limited availability of recordings from VENs themselves. In our current study, we utilized a transgenic mouse model to accurately locate the EVN, enabling us to conduct a substantial number of electrophysiological recordings from VENs. This approach has shed light on the normal electrophysiological characteristics of the VENs. Furthermore, we performed unilateral labyrinthectomy on mice. This procedure served as a model to simulate an acute vestibular deficit, allowing us to explore the participation of the VENs in this pathological state.

Our study of the VENs has yielded several significant findings. Firstly, we discovered that VENs exhibit spontaneous discharge accompanied by AHP. Building upon this, we proposed novel classification methods for VENs. Secondly, we revealed the expression of Kv4.2 and Kv4.3 channels in the VENs, which mediate the I_A_. In addition, we investigated the impact of common neurotransmitters on I_A_ in the VENs. Lastly, we have discovered the involvement of the VENs in the regulation of acute peripheral vestibular injury. Overall, our study provides a more comprehensive analysis of the normal electrophysiological characteristics of VENs and investigates their regulation under pathological conditions.

## Materials and Methods

### Electrophysiology

All animal handling and experimental procedures were reviewed and approved by the Animal Care and Use Committee of The Eye and ENT Hospital, Fudan University (Approval No: 2023DW195). To identify cholinergic VENs, we crossed homozygous ChAT-cre mice (JAX stock# 006410) with Rosa26-CAG-LSL-tdTomato mice (JAX stock# 007909) and obtained ChAT-cre-tdTomato mice, in which all cholinergic neurons are marked with red fluorescence throughout the brain. All newborn hybrid mice were genetically identified by PCR, and transgenic mice identified as positive were selected for subsequent studies. Acute VEN-containing brainstem slices were prepared from the brains of ChAT-cre-tdTomato mice of either sex from postnatal day (P) 12 to P14. Mice were anesthetized and decapitated. The brain was rapidly removed and immersed in pre-cooled (0–4°C) oxygenated high-sucrose artificial cerebrospinal fluid (aCSF) containing the following (in mmol/L): 230 sucrose, 10 D-glucose, 2.5 KCl, 3 MgCl_2_, 0.1 CaCl_2_, 1.25 NaH_2_PO_4_, 0.4 sodium L-ascorbate, 2 sodium pyruvate (Na-Py), and 3 myo-inositol, pH 7.4, oxygenated with 95% O_2_ and 5% CO_2_ mixed gas. Then, the brain was removed, and coronal EVN-containing brainstem slices (200 μm thick) were cut in ice-cold oxygenated high-sucrose aCSF on a vibratome (VT 1200 S, Leica, Germany). Then, these VEN-containing brainstem slices were incubated for at least 30 min in oxygenated standard recording aCSF at 34 °C, placed in a water bath prior to electrophysiological recording. The standard recording aCSF had the following composition (in mmol/L): 125 NaCl, 10 D-glucose, 2.5 KCl, 1.8 MgCl_2_, 1.2 CaCl_2_, 1.25 NaH_2_PO4, 0.4 sodium L-ascorbate, 2 Na-Py, and 3 myo-inositol, pH 7.4, oxygenated with 95% O_2_ and 5% CO_2_ mixed gas. A total of 83 neurons from 45 ChAT-cre-tdTomato mice were used for whole-cell patch-clamp recordings.

#### Drugs

QX-314 (5 mmol/L, Sigma-Aldrich, Saint Louis, MO, USA) was added to the intracellular solution to block voltage-gated Na^+^ channels. 4-aminopyridine (4-AP, 2 mmol/L, Sigma-Aldrich) and tetraethylammonium (TEA, 10 mmol/L, Sigma-Aldrich) were bath-applied *via* continuous perfusion in the extracellular solution to block the transient K^+^ current (I_A_) and the delayed rectifier K^+^ current (IK), respectively. Acetylcholine (ACh, 1 mmol/L, MedChemExpress, Monmouth Junction, NJ, USA) and calcitonin gene-related peptide (CGRP, 100 nmol/L, Aladdin, Shanghai, China), the main neurotransmitters in the vestibular afferent and efferent nervous system, were respectively bath-applied *via* continuous perfusion in the extracellular solution to evaluate the effect on the I_A_ current.

#### Electrophysiological Recordings and Analysis

All recordings (83 neurons from 45 transgenic mice) were performed on VEN-containing brainstem slices continuously superfused with oxygenated standard aCSF at room temperature (23–25 °C). Patch pipettes (5–6 MΩ) were pulled from borosilicate glass capillaries (BF150-86-10, Sutter Instruments, Novato, CA, USA) on a two-stage vertical pipette puller (Narishige, Tokyo, Japan). The patch solution contained the following (in mmol/L): 130 Potassium gluconate, 10 KCl, 2 EGTA, 10 HEPES, 10 creatine phosphate, 3 Mg-ATP, and 0.5 Na_2_-GTP (pH 7.3, 290 mOsm). 5 mmol/L QX-314 was added to the intercellular solution while recording the I_A_ current.

Whole-cell recordings were made from VENs visualized through a 63× water-immersion objective in an upright microscope (Olympus) equipped with a mercury lamp as the fluorescence excitation light source. The VENs were identified based on the self-referential, pronounced red fluorescence of the transgenic mice upon excitation with green light and their location.

The patch-clamp recordings were performed using an EPC10/2 amplifier (HEKA Electronics, Lambrecht Pfalz, Germany) driven by Patchmaster software (HEKA Electronics). VENs were held at − 90 mV, and the voltage-clamp recordings with an uncompensated series resistance < 10 MΩ were included in the analysis. Signals were filtered at 2 kHz and sampled at 100 kHz; the liquid junction potential of − 10 mV was corrected offline by subtracting 10 mV from all potentials.

The spontaneous firing patterns were analyzed from current-clamp recording (with no injected current). Resting membrane potential (RMP) and action potentials (APs) generated during the period of spontaneous activity were then averaged, and the following parameters were measured: threshold, amplitude, duration at half of its maximal height (half-width), rise slope, decay slope, and afterhyperpolarization (AHP) amplitude (AP threshold to trough of the AHP). The average interspike interval (ISI), coefficient of variation of ISI (CV_ISI_), and firing rate were calculated from at least 30 s of stable current-clamp recording without injected currents.

The I_A_ current was elicited by a step voltage-clamp recording protocol, consisting of a 200-ms pre-step at a holding potential of − 90 mV (to fully de-inactive I_A_) followed by voltage steps to potentials ranging from − 80 to +120 mV in 10-mV increments in order to measure the I_A_ peak amplitude and the current–voltage relationship (I–V curve); the peak amplitude of I_A_ was measured at the greatest current evoked after voltage activation. Besides, 10 mmol/L TEA and 5 mmol/L QX-314 were respectively added to the extracellular and intracellular solutions to subtract the IK and sodium current.

To obtain a steady-state activation curve (conductance–voltage/G–V relationship), the normalized conductance (G) was plotted against the test membrane potential (V_m_) and fitted with the Boltzmann equation: G/G_max_ = 1/1 + exp [(V_half_ − V_m_)/k], where G was calculated as follows: G = I/(V_m_ − V_rev_), yielding the membrane potential of the half-activation voltage (V_half_) and activation slope factor (k). Where V_rev_ is the reversal potential and G_max_ is the maximum chord conductance.

### Immunohistochemistry

ChAT-cre-tdTomato mice used for IHC experiments were anesthetized and transcardially perfused with phosphate-buffered saline (PBS; Sigma-Aldrich) and ice-cold 4% paraformaldehyde (PFA; Sigma-Aldrich). Each brain was quickly removed and post-fixed overnight at 4°C in the same fixative solution. Coronal brain slices (60 μm thick) were cut and collected as floating sections. The slices were blocked for 1 h at room temperature in a solution containing 0.6% Triton X-100 (Sigma-Aldrich) and 10% donkey serum (DS; Merck Millipore, Darmstadt, Germany) in PBS. After blocking, the slices were incubated with primary antibodies diluted in 5% DS and 0.3% Triton X-100 in PBS overnight at 4 °C. After three washes (30 min/each) in 0.3% Triton X-100 in PBS, the slices were incubated with the secondary antibodies diluted in 0.3 % Triton X-100 in PBS overnight at 4°C in the dark. Then, the sections were incubated with DAPI (500–800 μL, Beyotime, Shanghai, China) for 3–5 min after three washes in 0.3% Triton X-100 in PBS. Finally, the sections were mounted in Antifade Fluorescence Mounting Medium (Dako, Agilent Technologies, Inc.) and coverslipped. The following primary antibodies were used: Rabbit anti-ChAT (1:400, Sigma-Aldrich), Mouse anti-ChAT (1:400, Sigma-Aldrich), Rabbit anti-Kv4.3 (1:200, Alomone Labs, Jerusalem, Israel), Mouse anti-Kv4.2 (1:200, NeuroMab, Davis, CA, USA), and Rabbit anti-c-Fos (1:500, Santa Cruz, CA, USA). The following secondary antibodies were included: Alexa Fluor 488-Donkey anti-rabbit (1:500), Alexa Fluor 488-Donkey anti-mouse (1:500), Alexa Fluor 555-Donkey anti-rabbit (1:500), and Alexa Fluor 555-Donkey anti-mouse (1:500). All the above secondary antibodies were obtained from Jackson ImmunoResearch.

The sections were stored at − 20 °C, and immunostaining images were acquired using a Zeiss LSM 800 confocal microscope (Zeiss, Oberkochen, Germany) with 20× and 63× objectives. To visualize the VEN terminal fields, z-stack images were collected at a step size of 0.5 μm. Images were processed and analyzed with ImageJ (NIH Image) software. All the images shown are a single optical slice.

### Unilateral Labyrinthectomy (UL)

12 healthy P12 to 14 C57BL/6J mice were randomly divided into two groups: the UL group (*n* = 6) and the control group (*n* = 6). Following previously established methods [22], mice in the UL group underwent right-sided UL surgery under a microscope. After anesthesia, the mice were placed in the right lateral position. A postauricular incision was made to expose the external ear canal and tympanic bulla. After puncturing the tympanic membrane, the malleus and incus were removed, taking care to avoid damaging the stapedial artery. The oval window was enlarged, and a hook needle was used to mechanically disrupt the contents of the vestibular cavity, followed by irrigation with anhydrous ethanol for further chemical damage. The surgical void was filled with autologous fat, and the incision was sutured. A similar incision was made in the control group, and was subsequently sutured, without any other procedures. After recovery from anesthesia, the behavioral changes in mice were assessed using two tests: the open field test (OFT) and the air-righting reflex test. In the OFT, the mice were placed in a square open field box equipped with a camera and a light source. Their movements were recorded for 6 min, and the total distance traveled and average speed were measured to evaluate their locomotor activity. The air-righting reflex test involved dropping the mice supine from a height of 50 cm onto a padded surface. The mice underwent three trials, and their landing positions were scored. A score of 0 indicated a normal landing on all four limbs, a score of 1 indicated a moderate impairment with the mice landing on their side, and a score of 2 indicated a severe impairment with the mice landing on their back.

### Statistical Analyses

The numerical data are expressed as the mean ± standard error of the mean (SEM) with *P*-values of < 0.05, which is considered statistically significant. Data were analyzed using Igor Pro 6.22A software (WaveMetrics, Lake Oswego, OR, USA) and GraphPad Prism software 9.2.0 (GraphPad Software, Inc., CA, USA). Curve fitting was conducted using a sigmoidal fit (Boltzmann) in Origin 8 software (Origin Lab Corp.). The statistical significance of differences was assessed by either two-tailed paired *t* tests or two-way ANOVA test followed by Bonferroni multiple comparison tests. *, **, ***, **** indicate *P* < 0.05, 0.01, 0.001, and 0.0001, respectively.

## Results

### Electrophysiological Characteristics of Genetically Defined VENs

**Fig. 1:**
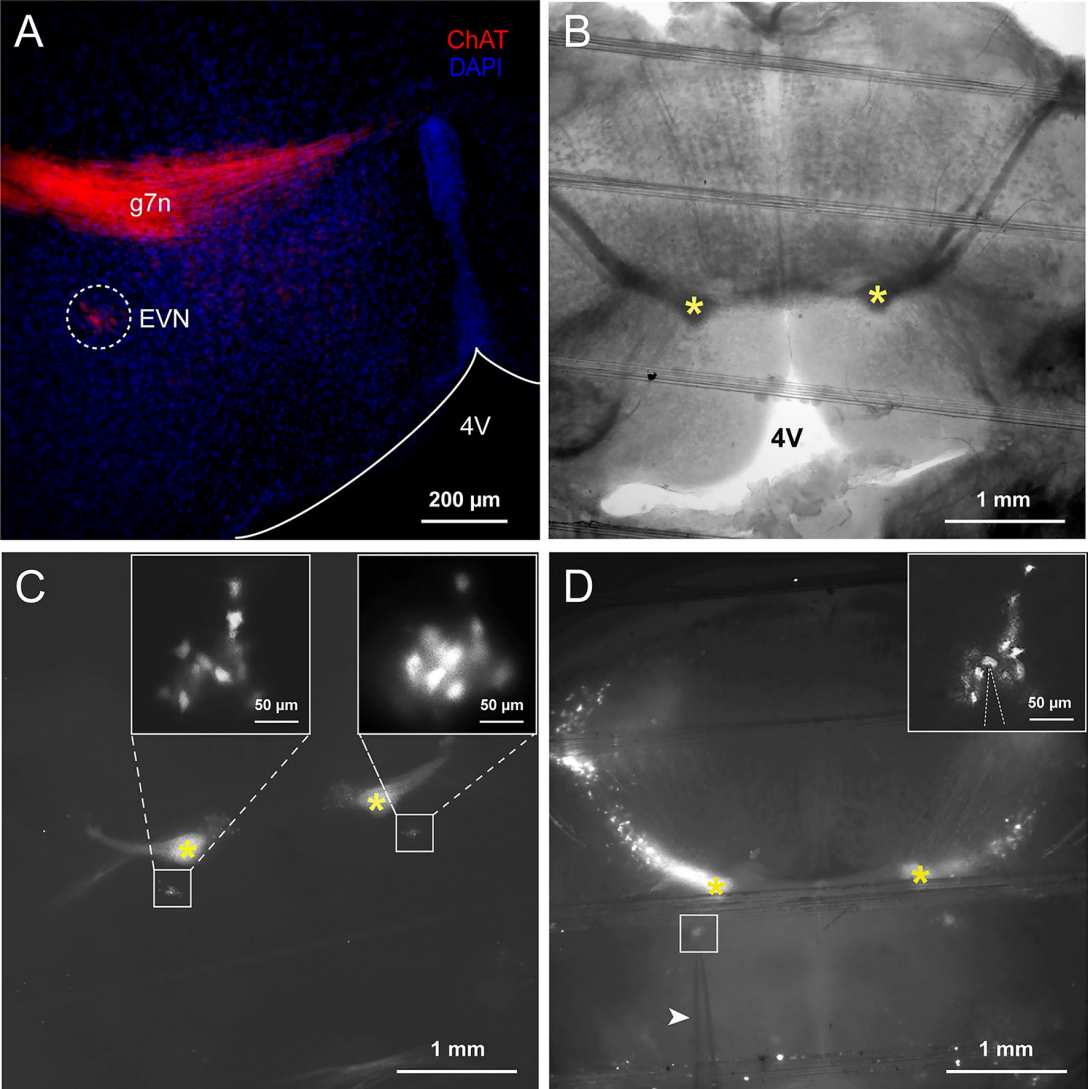
Identification of vestibular efferent neurons (VENs) in acute brainstem slices prepared from ChAT-cre-tdTomato mice. **A** Confocal image of a fixed brainstem section (40 μm) showing immunofluorescence staining of the efferent vestibular nucleus (EVN), double-stained for ChAT (red) and DAPI (blue). The dotted circle indicates the EVN. g7n: the genu of the facial nerve, 4V: the fourth ventricle. Scale bar, 200 μm. **B** DIC image of an acute brainstem slice containing the EVN (slice thickness: 200 μm, asterisks: g7n). **C** Fluorescence image of a bilateral brainstem slice showing the genu of the facial nerve bundle (asterisks: g7n) and VENs (white boxes; magnified views in insets). **D** Fluorescence image of a brainstem slice with a VEN under patch-clamp recording (white box; magnified view in inset). Arrowhead indicates the patch electrode. Scale bar, 1 mm.

In the mouse brainstem, the EVN is a small region with no visual boundaries, which makes VENs difficult to identify. We therefore took a genetic strategy that has proven to be successful and engineered ChAT-cre-tdTomato mice that allowed us to identify cholinergic VENs in the EVN. As shown in Fig. 1A, in fixed brainstem sections, extensive red fluorescence can be found in the genu of the facial nerve (g7n), which also consists of cholinergic neurons [13, 14]. More dorsolaterally, the EVN can be identified bilaterally as a small area of red fluorescence [8], ~ 0.7 mm from the midline, and − 5.8 mm caudal from Bregma [23]. We then prepared acute brainstem slices (Fig. 1B), and the EVN was similarly identified under fluorescence microscopy, and discrete VENs with red fluorescence were found under a 60 × objective (Fig. 1C) and subjected to patch-clamp recording (Fig. 1D).

**Fig. 2:**
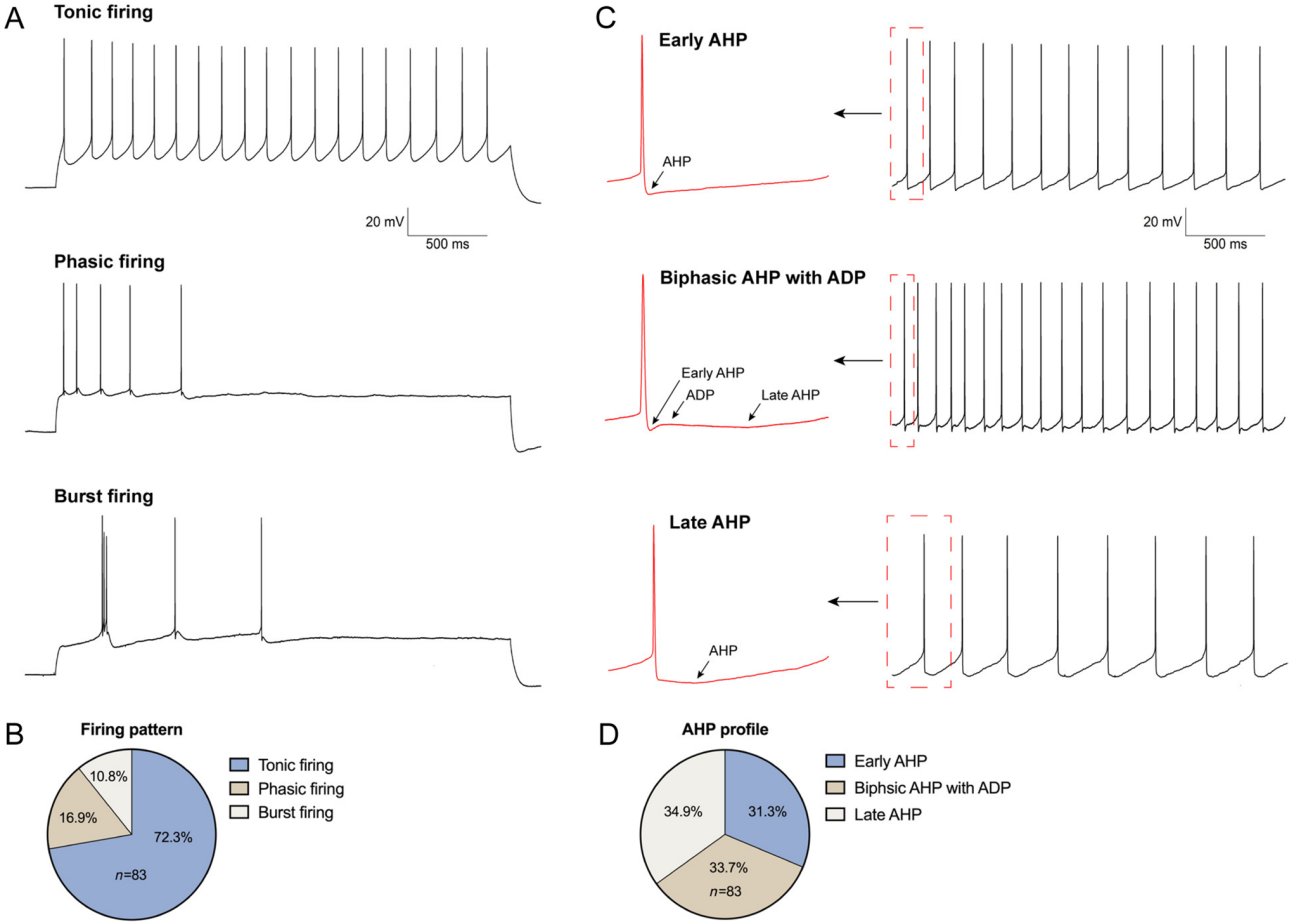
Classification of VENs based on characteristics of spiking pattern and afterhyperpolarization (AHP). **A** Representative voltage response of tonic, phasic, and burst firing VENs in response to step current injection under current-clamp. **B** Pie chart of the three spiking types (tonic, phasic, and burst) in all the VENs examined (*n* = 83). **C** Representative spikes showing an early AHP (top), a late AHP (bottom), and a biphasic AHP with an early AHP followed by a noticeable after depolarization (ADP) and a late AHP (middle). **D** Pie chart of the three AHP types in VENs.

In this study, we identified and recorded a total of 83 VENs in 45 transgenic mice aged between P12 and P14. Immediately following the success of obtaining a whole-cell recording, the cell was kept under current-clamp, and a negative current was injected to keep the cell at − 90 mV, to prevent the cell from firing spikes excessively between stimulations. To measure the RMP, we stepped the cell to zero current, and found that all VENs fired spikes spontaneously (Fig. 2). These VENs were heterogeneous in that they exhibited tonic, phasic, and burst firing of spikes (Fig. 2A and B). The vast majority of VENs (60/83) fired spikes regularly and repetitively, with a frequency ranging from 3 to 15 Hz. Phasic spiking VENs (14/83) fired 2 to 7 spikes only at the onset of zero current injection and remained silent afterwards. Lastly, burst spiking VENs fired spikes repetitively but in clusters; these made up a small percentage of all the neurons (9/83).

**Fig. 3:**
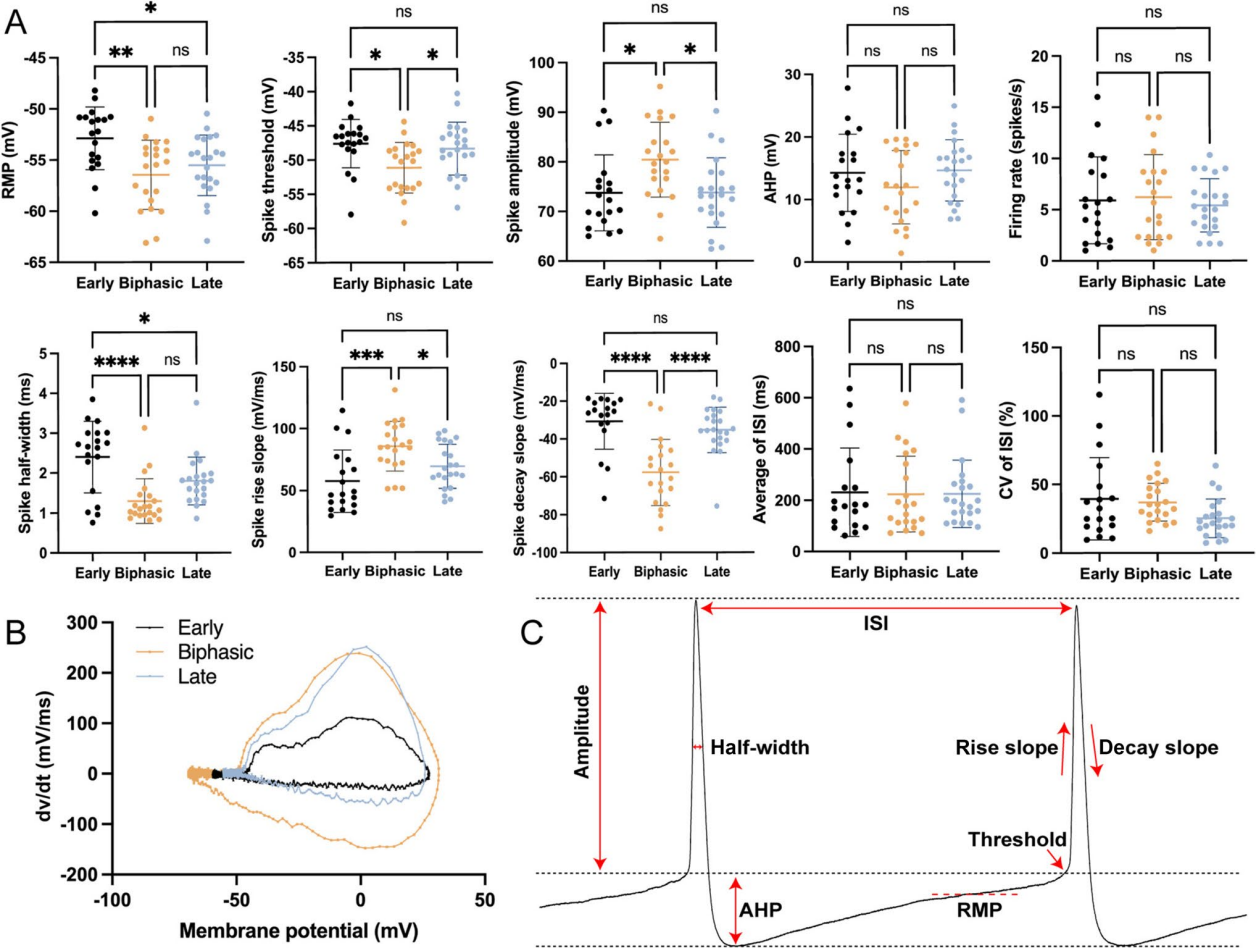
Comparison of electrophysiological properties among VENs of the three different AHP types. **A** Electrophysiological parameters of VENs, including resting membrane potential (RMP), kinetics of spikes (threshold, amplitude, rise slope, decay slope, half-width), spiking rate, average and coefficient of variation (CV) of inter-spike interval (ISI), AHP amplitude, from the three types of VENs with early AHP, biphasic AHP with ADP, and late AHP. **B** Plots of dv/dt for typical spikes from the three types of VENs. **C** Schematic diagram showing how electrophysiological parameters in A are determined from a typical spiking response. *P < 0.05, **P < 0.01, ***P < 0.001, ****P < 0.0001, ns *P* > 0.05.

Furthermore, following spike repolarization, three different shapes of AHP were found among different VENs (Fig. 2C and D), including an early AHP (29/83), a late AHP (28/83), and a biphasic AHP with after depolarization (26/83). Based on these spiking responses, we also calculated a range of electrophysiological properties for these VENs, which are listed in Table 1. Interestingly, we found significant differences in some, but not all, of the electrophysiological properties of VENs with different AHP types. Specifically, we performed statistical comparisons of the electrophysiological parameters related to spontaneous firing among the three groups of neurons: Early, Biphasic, and Late (Fig. 3A). First, we analyzed the differences in RMP and found that both the Biphasic and Late groups exhibited a significantly more hyperpolarized RMP compared to the Early group, whereas no significant difference was found between the Biphasic and Late groups. Next, we examined spike-related parameters. The threshold in the Biphasic group was significantly lower than that in the Early and Late groups, while the amplitude was significantly higher in the Biphasic group than in the other two groups. No significant differences were found between the Early and Late groups in either threshold or amplitude. Regarding half-width, the Early group exhibited a significantly longer duration compared to the Biphasic and Late groups, with no significant difference between the Biphasic and Late groups. In the analysis of spike kinetics, we found that both the rise slope and decay slope in the Biphasic group were significantly faster than those in the Early and Late groups, while no differences were found between the Early and Late groups for either slope. Comparisons of AHP amplitude revealed no significant differences among the three groups (Fig. 3A). Lastly, we assessed rhythmicity and excitability by analyzing firing rate, average ISI, and CV of ISI. No significant differences were detected among the three groups for any of these parameters. Plots of dV/dt from representative spikes of the three VEN subtypes revealed distinct differences in spike kinetics (Fig. 3B). A schematic illustration of how the electrophysiological parameters were extracted from a typical spike is provided in Fig. 3C. This suggests once again that VENs are composed of heterogeneous neurons, likely with different expression of voltage-gated Na^+^ and K^+^ channels.

**Table 1.** Electrophysiological parameters of spontaneous spiking in VENs (*n* = 83)

Parameters (*n* = 83)	Mean	SEM
Resting membrane potential (RMP)		
RMP (mV)	− 55.03	0.44
Action potential (AP)		
Threshold (mV)	− 49.06	0.50
Amplitude (mV)	76.01	1.01
Half-width (ms)	1.83	0.10
Rise slope (mV/ms)	71.16	3.00
Decay slope (mV/ms)	− 43.40	2.73
After hyperpolarization (mV)	13.61	0.72
Pacemaking		
ISI_AVG_ (ms)	233.67	20.09
CV_ISI_ (%)	34.91	2.95
Excitability		
Firing rate (spikes/s)	5.83	0.47

### A-type Potassium Channels in VENs and Their Modulation by ACh and CGRP

**Fig. 4:**
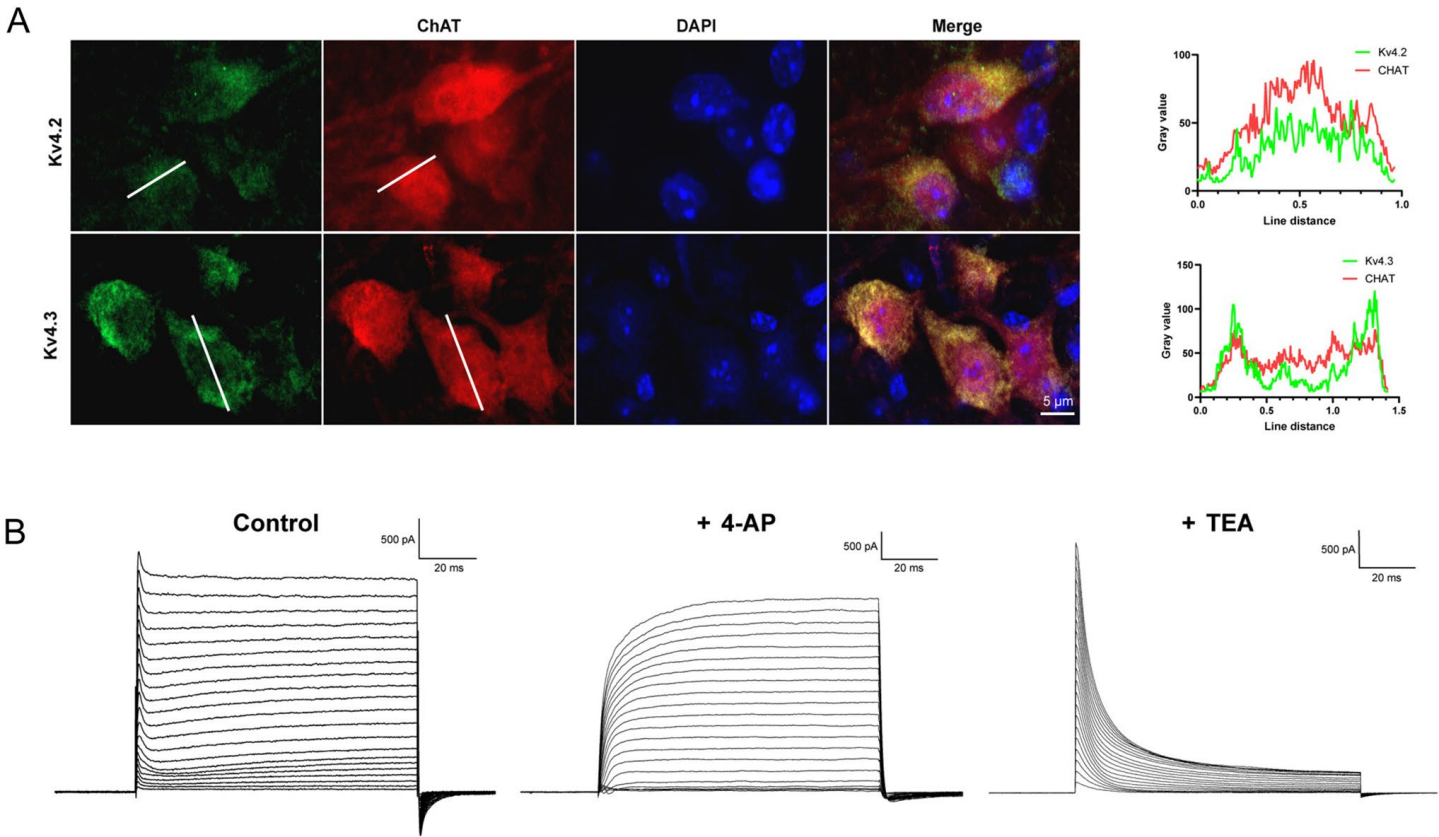
Colocalization of Kv4.2 and Kv4.3 with ChAT-positive neurons. **A** Left: Representative immunofluorescence images showing the expression of Kv4.2 (upper panels, green) and Kv4.3 (lower panels, green) in ChAT-positive neurons (red). Nuclei are counterstained with DAPI (blue). Merged images reveal colocalization of Kv4.2 and Kv4.3 with ChAT in neuronal cell bodies. White lines indicate the regions along which fluorescence intensity is measured for line scan analysis. Scale bar, 5 μm. Right: Fluorescence intensity profiles of Kv4.2 (upper graph, green) and Kv4.3 (lower graph, green) along the indicated white lines, compared with ChAT (red). The overlapping gray value peaks of Kv4.2 or Kv4.3 with ChAT suggest their co-expression in cholinergic neurons. **B** Whole-cell potassium currents in response to voltage steps recorded in VENs under voltage-clamp. Without blockers (Control), the current contained a slow-activating and sustained component and a quick-activating and transient component. The former is sensitive to TEA, and the latter is sensitive to 4-AP, the typical characteristics of A-type K^+^ current (I_A_).

The heterogeneity of VENs we described above is likely caused by different expression of voltage-gated K^+^ channels, presumably Kv4 that underlies the A-type K^+^ current [24]. Indeed, it has been shown that the Kv4 channels, including Kv4.2 and Kv4.3, are extensively expressed in vestibular nuclei [25]. In order to examine the expression of Kv4.2 and Kv4.3 in VENs, we used monoclonal antibodies against Kv4.2 or Kv4.3, and applied double immunofluorescence staining with antibodies against ChAT in fixed brainstem sections. We found that both channels were expressed abundantly in VENs (Fig. 4A). Colocalization analysis indicates that Kv4.2 and Kv4.3 are expressed in ChAT-positive cells and may contribute to the functional properties of cholinergic neurons (Fig. 4A).

We next examined the electrophysiological properties of currents that these channels underly. We performed patch-clamp recording in VENs and applied voltage steps under voltage-clamp. To isolate the K^+^ current, we included 5 mmol/L QX-314 in the pipette solution to block the Na^+^ current. As shown in Fig. 4B, we found VENs contained a total K^+^ current of a few nA, which consists of a slow-activating and non-inactivating component and a fast-activating and quickly inactivating component. While the former component was sensitive to 4-AP (2 mmol/L), the latter was sensitive to TEA (10 mmol/L), an indication that it is indeed an A-type K^+^ current [26].

**Fig. 5:**
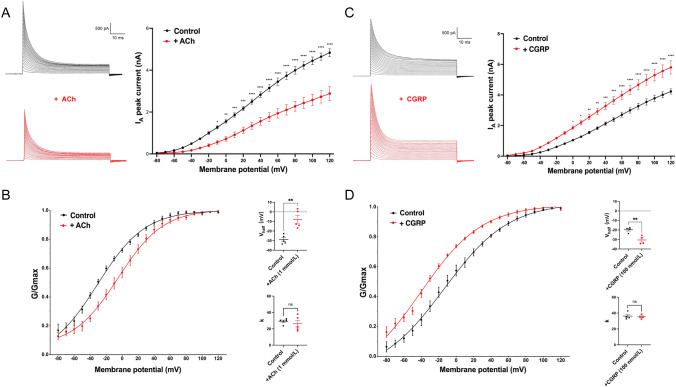
Modulation of I_A_ in VENs by acetylcholine (ACh) and calcitonin gene-related peptide (CGRP). **A** ACh decreases the peak amplitude of I_A_. **B** ACh shifts the steady-state activation curve (G-V curve) of I_A_ to the right. In the presence of ACh, V_half_ becomes more positive, while the slope k remains unchanged. **C** CGRP increases the peak amplitude of I_A_. **D** CGRP induces a leftward shift in the G-V curve of I_A_. In the presence of CGRP, V_half_ becomes more negative while the slope k is unchanged.

In the efferent vestibular system, acetylcholine (ACh) and calcitonin gene-related peptide (CGRP) are the major neurotransmitters [27], but it is not clear if and how they modulate the functions of VENs. To address this question, we examined the effect of ACh and CGRP on the A-type K^+^ current (I_A_) in VENs. We found that in the presence of ACh (1 mmol/L), I_A_ was greatly reduced in amplitude (Fig. 5A). Furthermore, we found that the activation curve of I_A_ was shifted to the right, and the half activation voltage (V_half_) became significantly more positive from − 28.93 ± 2.05 mV to − 8.00 ± 3.08 mV (*P* < 0.005, two-tailed paired *t* test). In contrast, CGRP (100 nmol/L) increased I_A_ in amplitude, shifted its activation to the left, and V_half_ became more negative, from − 19.79 ± 1.37 mV to − 30.52 ± 2.15 mV (*P* < 0.005, two-tailed paired *t* test). In neither of the two cases was the slope k changed significantly. Taken together, these results suggest spontaneous firing of VENs can be finely tuned by ACh and CGRP in opposite directions through their actions on I_A_ in these neurons.

### Modulation of VENs by Acute Vestibular Deprivation

**Fig. 6:**
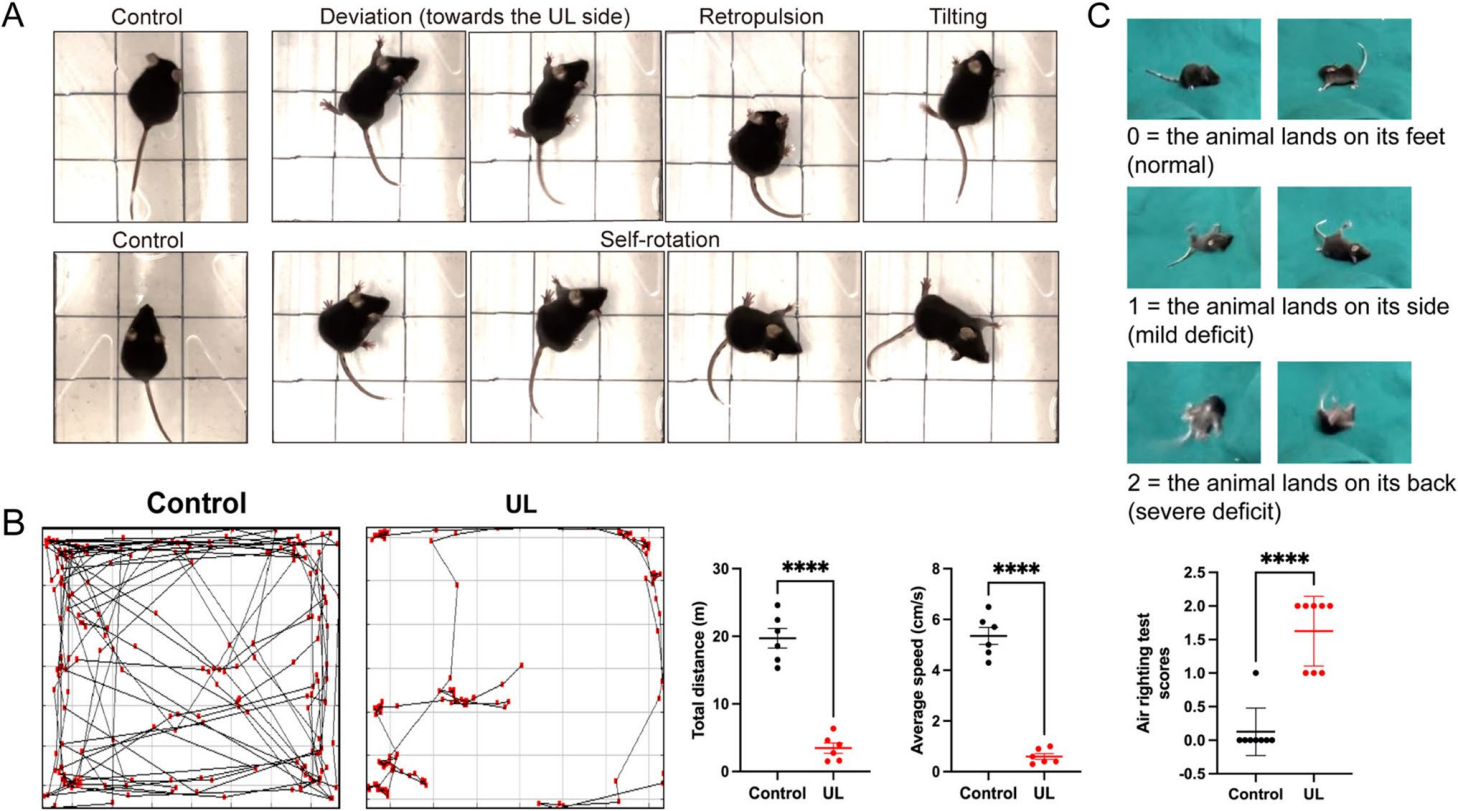
Vestibular dysfunctions in mice after unilateral labyrinthectomy (UL). **A** Mice in the UL group exhibit acute unilateral vestibular dysfunction, including deviation, retropulsion, tilting, and self-rotation (right panels). Mice in the Control group that were subjected to sham surgery behave normally (left panels). **B** Typical track plots in the open field test from the Control (left) and UL groups (right). Compared to the Control group, the UL group displays significantly impaired locomotor ability, as shown by the significant reduction in both the total distance moved and the average speed. **C** Air righting reflex test and scores. Compared to the Control group, the UL group displays a significantly impaired air righting reflex (lower panel).

In intact animals, vestibular deprivation not only leads to impaired vestibular function but also triggers compensation in the efferent vestibular system [28]. However, previous studies have been focused on the medial vestibular nucleus [29], and it is not clear if and how VENs are involved in the compensatory process. To address these questions, we performed a unilateral labyrinthectomy (UL) or sham surgery (Control) in ChAT-cre-tdTomato mice. After the UL, the mice exhibited severe symptoms of unilateral vestibular deficit, including abnormal posture and motor disturbances such as deviation towards the surgical side, retropulsion, tilting, and self-rotation (Fig. 6A). In the OFT, the UL mice demonstrated not only a significant reduction in the total distance travelled (19.71 ± 1.45 *vs* 3.48 ± 0.77 m, *P* < 0.0001, *n* = 6 per group) but also a decrease in the average speed (5.35 ± 0.34 *vs* 0.60 ± 0.12 cm/s, *P* < 0.0001, *n* = 6 per group) when compared to the Control group (Fig. 6B). Furthermore, in the air righting reflex test, the UL mice consistently exhibited an impaired reflex by landing on their side or back (Fig. 6C).

**Fig. 7:**
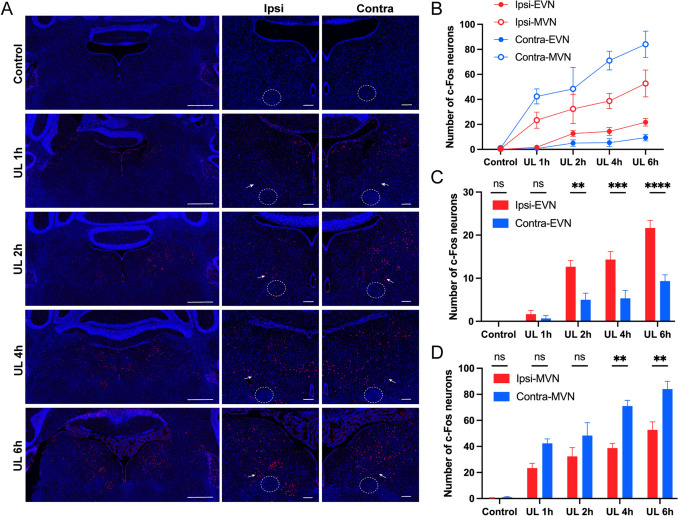
Activation of VENs is enhanced *in vivo* after UL. **A** Immunofluorescence staining of c-Fos in Control and at different times after UL. Left column: the overall view with 5× magnification; right two columns: the 10× magnified view of the ipsilateral and contralateral sides of UL, all double-stained for c-Fos (red) and DAPI (blue). No significant c-Fos positive VENs or vestibular afferent neurons (VANs) are observed in the Control group. After UL, VENs and VANs start to be activated at 2 h and 1 h post-UL surgery, respectively, with increasing activation levels over time up to 6 h after UL. Dashed circles indicate g7n; arrows indicate c-Fos-positive VENs. Scale bars, 500 and 100 μm. **B** The numbers of c-Fos positive VENs and VANs in bilateral EVN and MVN regions show a steady increase up to 6 h after UL. **C** Bar graph showing more c-Fos-positive VENs in the UL ipsilateral EVN region compared to the contralateral side, with statistically significant differences evident at 2, 4, and 6 h after UL. **D** Bar graph showing more c-Fos-positive VANs in the UL contralateral MVN region compared to the ipsilateral side, with statistically significant differences at 4 and 6 h after UL.

To probe functional changes in VENs following UL, we used immunofluorescence staining of c-Fos, an early gene product serving as a marker of neuronal activation [30]. We found that there was almost no expression of c-Fos in VENs in the EVN or vestibular afferent neurons (VANs) within the medial vestibular nucleus (MVN) in the Control group (*n* = 3). We then collected brains from the UL group at 1, 2, 4, and 6 h after surgery, and counted the number of c-Fos-positive VENs and VANs. As early as 1 h after UL, significantly more c-Fos-positive neurons were observed in the MVN region bilaterally, and the number continued to increase over the next 6 h. On the side contralateral to the UL, the number of c-Fos-positive VANs was significantly higher than that on the ipsilateral side at 4 and 6 h after UL (Fig. 7B and C, *n* = 3). In the EVN, more c-Fos-positive neurons were observed in the EVN region bilaterally starting from 2 h after UL. Interestingly, the number of c-Fos-positive neurons on the side ipsilateral to the UL was significantly higher than that on the contralateral side (Fig. 7B and D, *n* = 3).

**Fig. 8:**
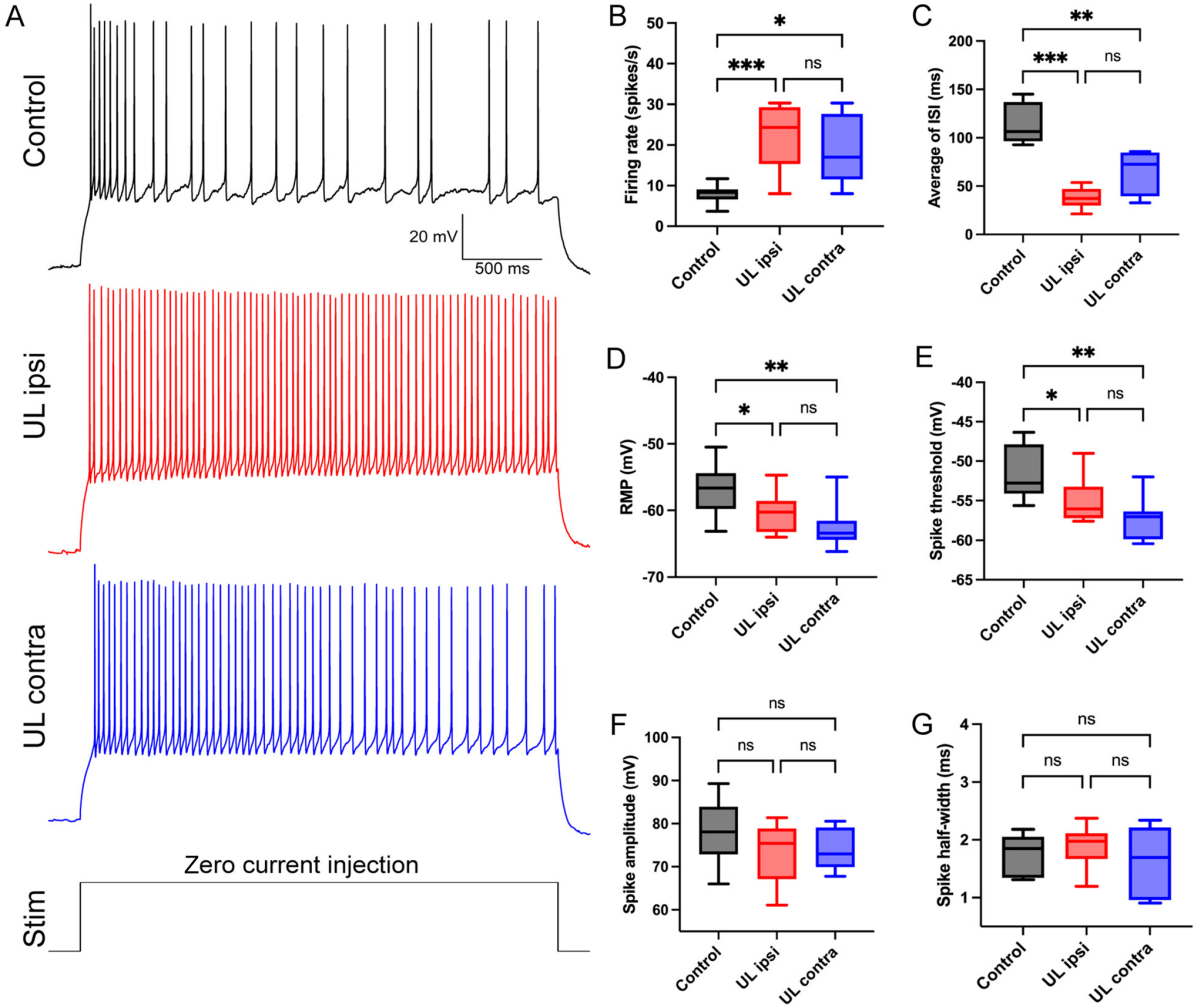
Changes in spontaneous firing and electrophysiological properties of bilateral VENs after UL. **A** Representative recordings of spontaneous firing in control, UL ipsilateral, and UL contralateral VENs. VENs are held under current-clamp and stepped to zero current. **B**–**G** Bar graphs showing statistical analyses of electrophysiological parameters (firing rate, average ISI, RMP, spike threshold, spike amplitude, spike half-width) in Control, UL ipsilateral, and UL contralateral VENs (*n* = 6 per group).

In order to determine if and how VENs are modulated by acute vestibular deprivation, we performed patch-clamp recording in VENs in both groups of mice. Although VENs are heterogeneous in spiking and AHP kinetics, there were no statistical differences in firing rate and ISI among them; we therefore combined them for statistical analysis. After behavioral tests, we prepared acute brainstem slices from the Control mice, as well as from the ipsilateral and contralateral sides of the UL mice. As described above, we recorded spontaneous firing by stepping the cell to zero current under current-clamp (Fig. 8A). We found a significant increase in firing rate in bilateral VENs following UL (UL ipsilateral *vs* Control, *P* < 0.001, UL contralateral *vs* Control: *P* < 0.05, *n* = 6 per group, Fig. 8B). Although the differences between bilateral VENs after UL were not significant, the mean firing rate on the UL ipsilateral side trended higher on the UL contralateral side. The ISI also displayed a similar trend of differences among the three groups (Fig. 8C). Furthermore, we found that both the RMP and the threshold of bilateral VENs were more negative compared to the Control group. Given that a more negative threshold increases excitability and that a more negative RMP reduces excitability, the increased excitability in VENs following UL is likely caused by the former, which outweighs the opposing effect caused by the latter (Fig. 8D and E). No statistically significant difference was found in the amplitude or half-width of the spike among the three groups, indicating that the spike kinetics were not significantly changed following UL (Fig. 8F and G).

## Discussion

In the current study, we have discovered that VENs exhibit the following typical characteristics: continuous spontaneous action potential discharges (spontaneous firing) in the absence of current injection, accompanied by AHP. Our findings contradict previous research, which reported the absence of spontaneous firing in VENs even with the application of current injection [13]. However, through extensive experiments and data analysis, our research has revealed that VENs exhibit significant spontaneous firing, similar to the electrophysiological properties recorded in afferent neurons of the MVN as reported by other researchers [1, 2]. This suggests that VENs are likely to participate in the functional modulation of the entire vestibular neural pathway through their intrinsic electrical activity.

### Subtypes of VENs

Next, we classified VENs based on electrophysiological parameters. On the one hand, VENs exhibit three distinct patterns of spike firing: tonic, phasic, and burst firing. Decoding and transmitting specific information relies heavily on neural firing patterns [31]. Tonic firing, the prevailing pattern recorded in VENs and which signifies a continuous and sustained discharge of action potentials, is associated with novelty-gated information storage [32, 33]. This firing mode may hint at the involvement of VENs in maintaining stable neural activity and regulating the integration and transmission of vestibular information. Phasic firing involves a transient and brief initial burst of action potentials in response to specific stimuli or events [34]. This firing pattern allows for rapid and precise responsiveness to changing sensory inputs, contributing to dynamic adjustments in posture and movement. Burst firing, characterized by a rapid succession of action potentials followed by a quiescent period [35], occurred less commonly in VENs. It may be associated with intense vestibular responses or the processing of sudden sensory stimuli.

On the other hand, the classification of VENs into three groups based on the AHP profiles in spontaneous firing has significant implications. Group A neurons exhibit an early AHP with a fast component, indicating rapid repolarization and potential involvement in fast and precise signaling. Group B neurons display a biphasic AHP with an early fast AHP, followed by an ADP and a late slow AHP. This profile suggests a complex response pattern, potentially involved in integrating and processing sensory inputs. Group C neurons show a late AHP with a slow component, possibly contributing to sustained neural activity and the maintenance of baseline firing. The distribution proportions of the three neuron groups within the EVN are similar. The main differences among these groups manifest in their RMP, threshold, amplitude, half-width, and rise/decay slope. Indeed, it is worth mentioning that similar classification methods have been applied to afferent neurons in the MVN [36], as well as the hypoglossal nucleus for horizontal gaze and the interstitial nucleus of Cajal for vertical and torsional gaze [37]. This similarity in classification suggests a potential coordination of electrical activity between vestibular afferent and efferent neurons at the central level of the brainstem. These interconnected neural circuits likely play a collective role in the control and maintenance of balance. Exploring the relationship between these neuron groups and their functional connectivity can provide valuable insights into the mechanisms of balance control and coordination.

### Modulation by Neurotransmitters

Through immunohistochemistry, we have determined the expression of Kv4.2 and Kv4.3 channels in VENs. Furthermore, we utilized patch-clamp techniques to confirm the distinct electrophysiological phenotype of I_A_. As common channels involved in neuronal excitability, our research group has a longstanding interest in I_A_. In previous studies, we found changes in the expression of Kv4.2 and Kv4.3 in the EVN following peripheral vestibular deficit and stimulation in rats. These findings provide evidence for the potential involvement of Kv4.2 and Kv4.3 in the regulation of excitability. We further applied two widely recognized neurotransmitters in the vestibular system, CGRP and ACh, which can work together to modulate vestibular sensory function [38]. ACh enhances excitability and promotes tonic firing by activating muscarinic ACh receptors, which close K^+^ channels [39]. CGRP plays a potential role in the pathophysiology of vestibular migraine [40], and down-regulating CGRP levels or inhibiting its activity may offer therapeutic benefits in improving vestibular dysfunction [41].

The results demonstrate that the addition of ACh leads to a downward shift in the I–V curve, with a more pronounced decrease as the stimulus voltage increases. This suggests that ACh has a voltage-dependent effect on the I_A_ peak, causing its attenuation. The activation curve shows that the addition of ACh shifts the G–V curve to the right, correcting the V_half_, indicating that ACh raises the activation voltage of I_A_. On the other hand, the addition of CGRP exhibits the opposite trend, with an upward shift in the I–V curve and a leftward shift in the G-V curve. The more negative V_half_ suggests that CGRP facilitates the activation of I_A_ at lower voltages. Based on previous research findings, it has been suggested that down-regulation of I_A_ in pyramidal neuron dendrites can increase neuronal excitability [42]. Therefore, in light of our results, we hypothesize that ACh may enhance the excitability of the EVN by down-regulating the peak amplitude of I_A_ and delaying its activation. Conversely, CGRP may decrease excitability by producing the opposite effect. Our findings suggest that neurotransmitter activity can significantly affect I_A_, potentially playing a role in the regulation of neuronal excitability. In summary, ACh and CGRP interact closely in the EVN, serving as co-transmitters that shape vestibular neuron activity. They are co-expressed in the same efferent neurons and co-released onto vestibular end-organs [11, 43]. ACh provides fast synaptic actions *via* nicotinic receptors and slower modulatory actions *via* muscarinic receptors, while CGRP fine-tunes these effects by prolonging excitation and up-regulating receptor responsiveness [44, 45]. Both signals converge on the modulation of ion channels (such as A-type K^+^ and M-type K^+^ currents) that govern neuronal excitability, resulting in enhanced firing of vestibular afferents [43]. These interactions are not only critical for moment-to-moment synaptic transmission but also have important implications for plasticity: ACh and CGRP together facilitate vestibular compensation and adaptation of reflexes after perturbations. While the precise mechanisms are still being unraveled, current peer-reviewed studies indicate that CGRP amplifies cholinergic vestibular efferent signaling and that both co-transmitters contribute to the dynamic regulation and recovery of vestibular function [11]. This dual-transmitter system exemplifies how modulatory neurotransmitters can cooperate to influence neural circuit plasticity, and it presents potential targets (e.g., muscarinic, nicotinic, and CGRP receptors) for interventions to improve vestibular compensation in clinical settings.

### Modulation by Sensory Deprivation

Previous studies have demonstrated that the MVN actively participates in vestibular compensation. Immunofluorescence analysis has revealed an upregulation of c-Fos expression in bilateral MVN neurons following UL, indicating their activation [29]. Electrophysiological recordings further support these findings, showing a significant reduction in excitability of MVN neurons on the side ipsilateral to the UL, while neurons in the contralateral MVN exhibit a marked increase in sensitivity to electrical stimulation [46]. However, to date, the role of the EVN in acute peripheral vestibular deficit remains unexplored. Hence, our study aimed to investigate the activation and involvement of VENs following UL. Through a comprehensive analysis combining immunofluorescence and electrophysiology, we observed a gradual increase in c-Fos expression in VENs in response to acute pathological conditions. These neurons exhibited rapid and sustained activation, which began within 2 h after UL and persisted over time. Moreover, they displayed enhanced intrinsic excitability, characterized by an elevation in spontaneous firing rate, a decrease in RMP, and a lowered threshold for activation. Notably, the activation and heightened excitability were more pronounced on the ipsilateral side compared to the contralateral side of the UL, exhibiting a trend distinct from that in MVN neurons. Collectively, these findings suggest the involvement of the EVN in the regulation and compensation mechanisms following acute peripheral vestibular deficit.

### Involvement of VENs in Compensation After Sensory Deprivation

Studies indicate that VENs provide bilateral, predominantly contralateral, innervation to the peripheral vestibular system, primarily exerting an inhibitory effect on type II hair cell input [8, 47]. Based on these findings and our experimental data, we propose that VENs may play a crucial role in early vestibular compensation following unilateral vestibular peripheral deprivation. Mechanistically, the ipsilateral VENs exhibit heightened excitability, intensifying their inhibitory input to contralateral type II hair cells. This strengthened inhibition reduces the excitability of the contralateral peripheral vestibular system, thereby decreasing the imbalance in firing rates between the silenced ipsilateral and active contralateral vestibular inputs, contributing to an improvement in the symptoms of unilateral balance deficit. Evidence from non-mammalian models, such as turtles, demonstrates that efferent stimulation can rapidly excite calyx/dimorphic afferents through the activation of α4β2-containing nAChRs, leading to direct depolarization of calyceal endings. While direct evidence in mammals is limited, the presence of α4β2 subunit transcripts in mammalian vestibular ganglia suggests a potential for similar excitatory mechanisms [48]. Recent research has shown that mAChRs are expressed on vestibular afferent terminals and play a role in modulating their excitability. Activation of these receptors can lead to slow excitation of afferents, potentially through the modulation of ion channels such as KCNQ (Kv7) channels. This slow excitation complements the rapid inhibitory effects mediated by α9-containing nAChRs on hair cells, highlighting the complexity of cholinergic modulation in the vestibular system [49].

During the acute phase following UL, VENs on both sides exhibited increased neuronal excitability, as evidenced by accelerated spontaneous firing rates, elevated input resistance, and suppression of outward K^+^ currents. Notably, these excitability-enhancing changes were more pronounced in VENs on the ipsilateral side compared to the contralateral side. In light of previous studies on VANs after UL, we propose a hypothesis for the brainstem-level response mechanism during the acute phase of vestibular compensation. Following UL, the peripheral primary vestibular input is disrupted, resulting in the loss of excitatory drive to VANs in the ipsilateral MVN. This leads to a reduction in VAN excitability on the lesioned side [50]. As a compensatory response—likely mediated by synaptic and intrinsic plasticity—VENs in the ipsilateral EVN exhibit increased excitability to counterbalance the loss of afferent input within the vestibular circuitry. Given that reciprocal inhibitory interactions exist between the bilateral MVNs, the disruption of excitatory input to ipsilateral VANs may disturb the balance of electrical activity between both sides. In the absence of ipsilateral VAN-mediated inhibitory input, VANs on the contralateral side exhibit increased excitability [51]. We hypothesize that a similar bilateral balance mechanism may exist within the EVN. With the enhanced excitability of ipsilateral VENs, contralateral VENs may also increase their activity to maintain bilateral symmetry. However, the excitability increase in contralateral VENs appears to be less prominent, possibly due to inhibitory influences from the hyperactive contralateral VANs on the same-side VENs. Together, these findings suggest the existence of a rapid, closed-loop response circuit between vestibular afferent and efferent components at the brainstem level during the acute phase post-UL, which contributes to the early stages of vestibular compensation.

The limitations of our study lie in its primary focus on the role of VENs in the acute phase, with insufficient exploration of their involvement in the chronic phase. In future studies, we will extend the post-UL recording window to clarify the mechanisms of VENs in long-term chronic compensation. In addition, we did not investigate the effects of different concentrations of neurotransmitter drugs on current changes. We plan to incorporate a wider range of concentration gradients to explore the specific effects of neurotransmitter drugs in future experiments.

Through this study, we have explored the normal electrophysiological activity of VENs and their regulatory functional changes under altered external conditions. Our results highlight the role of Kv4.2 and Kv4.3 channels in regulating neuronal excitability and suggest their involvement in the mechanism underlying neuronal activation in the EVN. Notably, this study marks the first discovery of participation of the EVN in the regulation and compensation processes following acute peripheral vestibular deficit. These findings provide data support for potential mechanisms underlying the involvement of VENs in compensation. Furthermore, our research results offer methods and insights for future investigations on the role of VENs as novel therapeutic targets in clinical settings. By delving deeper into the electrophysiological activity of VENs, we can gain a better understanding of their involvement in functions such as balance and spatial orientation, and guide the development of treatment strategies for vestibular system-related disorders.

## References

[1] McCall AA, Miller DM, Yates BJ. Descending influences on vestibulospinal and vestibulosympathetic reflexes. Front Neurol 2017, 8: 112. 28396651 10.3389/fneur.2017.00112 PMC5366978

[2] Mackrous I, Carriot J, Cullen KE. Context-independent encoding of passive and active self-motion in vestibular afferent fibers during locomotion in Primates. Nat Commun 2022, 13: 120. 35013266 10.1038/s41467-021-27753-z PMC8748921

[3] Pieter Medendorp W, Selen LJP. Vestibular contributions to high-level sensorimotor functions. Neuropsychologia 2017, 105: 144–152. 28163007 10.1016/j.neuropsychologia.2017.02.004

[4] Zhou L, Gu Y. Cortical mechanisms of multisensory linear self-motion perception. Neurosci Bull 2023, 39: 125–137. 35821337 10.1007/s12264-022-00916-8 PMC9849545

[5] Khan S, Chang R. Anatomy of the vestibular system: A review. NeuroRehabilitation 2013, 32: 437–443. 23648598 10.3233/NRE-130866

[6] Cullen KE. The vestibular system: Multimodal integration and encoding of self-motion for motor control. Trends Neurosci 2012, 35: 185–196. 22245372 10.1016/j.tins.2011.12.001 PMC4000483

[7] Highstein SM, Holstein GR. The anatomy of the vestibular nuclei. Prog Brain Res 2006, 151: 157–203. 16221589 10.1016/S0079-6123(05)51006-9

[8] Lorincz D, Poppi LA, Holt JC, Drury HR, Lim R, Brichta AM. The long and winding road-vestibular efferent anatomy in mice. Front Neural Circuits 2022, 15: 751850. 35153679 10.3389/fncir.2021.751850 PMC8832101

[9] Meredith GE. Comparative view of the central organization of afferent and efferent circuitry for the inner ear. Acta Biol Hung 1988, 39: 229–249. 3077006

[10] Cullen KE, Wei RH. Differences in the structure and function of the vestibular efferent system among vertebrates. Front Neurosci 2021, 15: 684800. 34248486 10.3389/fnins.2021.684800 PMC8260987

[11] Mathews MA, Camp AJ, Murray AJ. Reviewing the role of the efferent vestibular system in motor and vestibular circuits. Front Physiol 2017, 8: 552. 28824449 10.3389/fphys.2017.00552 PMC5539236

[12] Lorincz D, Drury HR, Lim R, Brichta AM. Immunohistochemical identification of sensory neuropeptides calcitonin gene-related peptide, substance P, and pituitary adenylate cyclase-activating polypeptide in efferent vestibular nucleus neurons. Neuroendocrinology 2025, 115: 269–282. 39662068 10.1159/000542984 PMC11991750

[13] Leijon S, Magnusson AK. Physiological characterization of vestibular efferent brainstem neurons using a transgenic mouse model. PLoS One 2014, 9: e98277. 24867596 10.1371/journal.pone.0098277 PMC4035287

[14] Mathews MA, Murray A, Wijesinghe R, Cullen K, Tung VWK, Camp AJ. Efferent vestibular neurons show homogenous discharge output but heterogeneous synaptic input profile *in vitro*. PLoS One 2015, 10: e0139548. 26422206 10.1371/journal.pone.0139548 PMC4589407

[15] Rudy B, Maffie J, Amarillo Y, Clark B, Goldberg EM, Jeong HY, *et al*. Voltage gated potassium channels: Structure and function of Kv1 to Kv9 subfamilies. Encyclopedia of Neuroscience. Amsterdam: Elsevier, 2009: 397–425.

[16] Birnbaum SG, Varga AW, Yuan LL, Anderson AE, David Sweatt J, Schrader LA. Structure and function of Kv4-family transient potassium channels. Physiol Rev 2004, 84: 803–833. 15269337 10.1152/physrev.00039.2003

[17] Serrano-Novillo C, Oliveras A, Ferreres JC, Condom E, Felipe A. Remodeling of Kv7.1 and Kv7.5 Expression in Vascular Tumors. Int J Mol Sci 2020, 21: 6019. 32825637 10.3390/ijms21176019 PMC7503939

[18] Coetzee WA, Amarillo Y, Chiu J, Chow A, Lau D, McCormack T. Molecular diversity of K+ channels. Ann N Y Acad Sci 1999, 868: 233–285. 10414301 10.1111/j.1749-6632.1999.tb11293.x

[19] Huang HY, Cheng JK, Shih YH, Chen PH, Wang CL, Tsaur ML. Expression of A-type K channel alpha subunits Kv 4.2 and Kv 4.3 in rat spinal lamina II excitatory interneurons and colocalization with pain-modulating molecules. Eur J Neurosci 2005, 22: 1149–1157. 16176357 10.1111/j.1460-9568.2005.04283.x

[20] Carrasquillo Y, Nerbonne JM. IA channels: Diverse regulatory mechanisms. Neuroscientist 2014, 20: 104–111. 24106264 10.1177/1073858413504003 PMC10158498

[21] Peraza DA, Cercós P, Miaja P, Merinero YG, Lagartera L, Socuéllamos PG, *et al*. Identification of IQM-266, a novel DREAM ligand that modulates K_V_4 currents. Front Mol Neurosci 2019, 12: 11. 30787866 10.3389/fnmol.2019.00011 PMC6373780

[22] Simon F, Pericat D, Djian C, Fricker D, Denoyelle F, Beraneck M. Surgical techniques and functional evaluation for vestibular lesions in the mouse: Unilateral labyrinthectomy (UL) and unilateral vestibular neurectomy (UVN). J Neurol 2020, 267: 51–61. 32556569 10.1007/s00415-020-09960-8 PMC7718198

[23] Paxinos G, Franklin KBJ. Paxinos and Franklin's the Mouse Brain in Stereotaxic Coordinates. 5^th^ edn. Academic Press, 2019.

[24] Jerng HH, Pfaffinger PJ, Covarrubias M. Molecular physiology and modulation of somatodendritic A-type potassium channels. Mol Cell Neurosci 2004, 27: 343–369. 15555915 10.1016/j.mcn.2004.06.011

[25] Serôdio P, Rudy B. Differential expression of Kv4 K+ channel subunits mediating subthreshold transient K+ (A-type) currents in rat brain. J Neurophysiol 1998, 79: 1081–1091. 9463463 10.1152/jn.1998.79.2.1081

[26] Hu CL, Liu Z, Zeng XM, Liu ZQ, Chen XH, Zhang ZH, *et al*. 4-aminopyridine, a Kv channel antagonist, prevents apoptosis of rat cerebellar granule neurons. Neuropharmacology 2006, 51: 737–746. 16806301 10.1016/j.neuropharm.2006.05.013

[27] Schneider GT, Lee C, Sinha AK, Jordan PM, Holt JC. The mammalian efferent vestibular system utilizes cholinergic mechanisms to excite primary vestibular afferents. Sci Rep 2021, 11: 1231. 33441862 10.1038/s41598-020-80367-1 PMC7806594

[28] Lacour M, Helmchen C, Vidal PP. Vestibular compensation: The neuro-otologist’s best friend. J Neurol 2016, 263: S54–S64. 27083885 10.1007/s00415-015-7903-4 PMC4833803

[29] Wang J, Tian E, Zhang Y, Guo Z, Chen J, Kong W, *et al*. The effects of unilateral labyrinthectomy on monoamine neurotransmitters in the medial vestibular nucleus of rats. Biomolecules 2023, 13: 1637. 38002319 10.3390/biom13111637 PMC10669524

[30] Cai G, Lu Y, Chen J, Yang D, Yan R, Ren M, *et al*. Brain-wide mapping of c-Fos expression with fluorescence micro-optical sectioning tomography in a chronic sleep deprivation mouse model. Neurobiol Stress 2022, 20: 100478. 35991686 10.1016/j.ynstr.2022.100478 PMC9389418

[31] Shao J, Liu Y, Gao D, Tu J, Yang F. Neural burst firing and its roles in mental and neurological disorders. Front Cell Neurosci 2021, 15: 741292. 34646123 10.3389/fncel.2021.741292 PMC8502892

[32] Davis OC, Dickie AC, Mustapa MB, Boyle KA, Browne TJ, Gradwell MA, *et al*. Calretinin-expressing islet cells are a source of pre- and post-synaptic inhibition of non-peptidergic nociceptor input to the mouse spinal cord. Sci Rep 2023, 13: 11561. 37464016 10.1038/s41598-023-38605-9 PMC10354228

[33] Grace AA, Floresco SB, Goto Y, Lodge DJ. Regulation of firing of dopaminergic neurons and control of goal-directed behaviors. Trends Neurosci 2007, 30: 220–227. 17400299 10.1016/j.tins.2007.03.003

[34] Dougherty PM, Chen J. Relationship of membrane properties, spike burst responses, laminar location, and functional class of dorsal horn neurons recorded *in vitro*. J Neurophysiol 2016, 116: 1137–1151. 27334950 10.1152/jn.00187.2016 PMC5013171

[35] Ferrari FS, Viana RL, Lopes SR, Stoop R. Phase synchronization of coupled bursting neurons and the generalized Kuramoto model. Neural Netw 2015, 66: 107–118. 25828961 10.1016/j.neunet.2015.03.003

[36] Saito Y, Takazawa T, Ozawa S. Relationship between afterhyperpolarization profiles and the regularity of spontaneous firings in rat medial vestibular nucleus neurons. Eur J Neurosci 2008, 28: 288–298. 18702700 10.1111/j.1460-9568.2008.06338.x

[37] Saito Y, Sugimura T, Yanagawa Y. Comparisons of neuronal and excitatory network properties between the rat brainstem nuclei that participate in vertical and horizontal gaze holding. eNeuro 2017, 4: ENEURO.0180–17.2017. 10.1523/ENEURO.0180-17.2017 PMC5616193 28966973

[38] Luebke AE, Holt JC, Jordan PM, Wong YS, Caldwell JS, Cullen KE. Loss of α-calcitonin gene-related peptide (αCGRP) reduces the efficacy of the Vestibulo-ocular Reflex (VOR). J Neurosci 2014, 34: 10453–10458. 25080603 10.1523/JNEUROSCI.3336-13.2014 PMC4115147

[39] Metherate R, Cox CL, Ashe JH. Cellular bases of neocortical activation: Modulation of neural oscillations by the nucleus basalis and endogenous acetylcholine. J Neurosci 1992, 12: 4701–4711. 1361197 10.1523/JNEUROSCI.12-12-04701.1992 PMC6575759

[40] Hoskin JL, Fife TD. New anti-CGRP medications in the treatment of vestibular migraine. Front Neurol 2022, 12: 799002. 35153979 10.3389/fneur.2021.799002 PMC8828914

[41] Zhang Y, Zhang Y, Tian K, Wang Y, Fan X, Pan Q, *et al*. Calcitonin gene-related peptide facilitates sensitization of the vestibular nucleus in a rat model of chronic migraine. J Headache Pain 2020, 21: 72. 32522232 10.1186/s10194-020-01145-y PMC7288551

[42] Liu YQ, Huang WX, Sanchez RM, Min JW, Hu JJ, He XH, *et al*. Regulation of Kv4.2 A-type potassium channels in HEK-293 cells by hypoxia. Front Cell Neurosci 2014, 8: 329. 25352783 10.3389/fncel.2014.00329 PMC4196569

[43] Lee C, Jones TA. Neuropharmacological targets for drug action in vestibular sensory pathways. J Audiol Otol 2017, 21: 125–132. 28942632 10.7874/jao.2017.00171 PMC5621797

[44] Chris Holt J, Jordan PM, Lysakowski A, Shah A, Barsz K, Contini D. Muscarinic acetylcholine receptors and M-currents underlie efferent-mediated slow excitation in *Calyx*-bearing vestibular afferents. J Neurosci 2017, 37: 1873–1887. 28093476 10.1523/JNEUROSCI.2322-16.2017 PMC5320615

[45] Wang X, Shi Z, Xue J, Zhang L, Feng L, Zhang Z. Expression of calcitonin gene-related peptide in efferent vestibular system and vestibular nucleus in rats with motion sickness. PLoS One 2012, 7: e47308. 23056625 10.1371/journal.pone.0047308 PMC3467246

[46] Xue WX, Li QX, Zhang YX, Zhang XY, Yung WH, Wang JJ, *et al*. Changes in sensitivity of bilateral medial vestibular nuclear neurons responding to input stimuli during vestibular compensation and the underlying ionic mechanism. Sheng Li Xue Bao 2022, 74: 135–144. 35503061

[47] Yu Z, Michael McIntosh J, Sadeghi SG, Glowatzki E. Efferent synaptic transmission at the vestibular type II hair cell synapse. J Neurophysiol 2020, 124: 360–374. 32609559 10.1152/jn.00143.2020 PMC7500374

[48] Chris Holt J, Kewin K, Jordan PM, Cameron P, Klapczynski M, Michael McIntosh J, *et al*. Pharmacologically distinct nicotinic acetylcholine receptors drive efferent-mediated excitation in *Calyx*-bearing vestibular afferents. J Neurosci 2015, 35: 3625–3643. 25716861 10.1523/JNEUROSCI.3388-14.2015 PMC4339364

[49] Ramakrishna Y, Manca M, Glowatzki E, Sadeghi SG. Cholinergic modulation of membrane properties of *Calyx* terminals in the vestibular periphery. Neuroscience 2021, 452: 98–110. 33197502 10.1016/j.neuroscience.2020.10.035 PMC8054478

[50] Chen ZP, Zhang XY, Peng SY, Yang ZQ, Wang YB, Zhang YX, *et al*. Histamine H1 receptor contributes to vestibular compensation. J Neurosci 2019, 39: 420–433. 30413645 10.1523/JNEUROSCI.1350-18.2018 PMC6335742

[51] Fetter M. Acute unilateral loss of vestibular function. Handb Clin Neurol 2016, 137: 219–229. 27638073 10.1016/B978-0-444-63437-5.00015-7

